# The lack of temporal brain dynamics asymmetry as a signature of impaired consciousness states

**DOI:** 10.1098/rsfs.2022.0086

**Published:** 2023-04-14

**Authors:** Elvira G-Guzmán, Yonatan Sanz Perl, Jakub Vohryzek, Anira Escrichs, Dragana Manasova, Başak Türker, Enzo Tagliazucchi, Morten Kringelbach, Jacobo D. Sitt, Gustavo Deco

**Affiliations:** ^1^ Department of Information and Communication Technologies, Centre for Brain and Cognition, Computational Neuroscience Group, Universitat Pompeu Fabra, Barcelona, Spain; ^2^ Sorbonne Université, Institut du Cerveau - Paris Brain Institute - ICM, Inserm Physiological Investigation of Clinically Normal and Impaired Cognition Team, CNRS, 75013, Paris, France; ^3^ Centre for Eudaimonia and Human Flourishing, Linacre College, University of Oxford, Oxford, UK; ^4^ Université Paris Cité, Paris, France; ^5^ Buenos Aires Physics Institute and Physics Department, University of Buenos Aires, Buenos Aires, Argentina; ^6^ Latin American Brain Health (BrainLat), Universidad Adolfo Ibáñez, Santiago, Chile; ^7^ Department of Clinical Medicine, Center for Music in the Brain, Aarhus University, Jutland, Denmark; ^8^ Institució Catalana de la Recerca i Estudis Avançats (ICREA), Barcelona, Catalonia, Spain; ^9^ Department of Neuropsychology, Max Planck Institute for human Cognitive and Brain Sciences, Leipzig, Germany; ^10^ School of Psychological Sciences, Monash University, Melbourne, Australia

**Keywords:** reversibility, disorder of consciousness, functional magnetic resonance imaging, non-equilibrium, temporal asymmetry, arrow of time

## Abstract

Life is a constant battle against equilibrium. From the cellular level to the macroscopic scale, living organisms as dissipative systems require the violation of their detailed balance, i.e. metabolic enzymatic reactions, in order to survive. We present a framework based on temporal asymmetry as a measure of non-equilibrium. By means of statistical physics, it was discovered that temporal asymmetries establish an arrow of time useful for assessing the reversibility in human brain time series. Previous studies in human and non-human primates have shown that decreased consciousness states such as sleep and anaesthesia result in brain dynamics closer to the equilibrium. Furthermore, there is growing interest in the analysis of brain symmetry based on neuroimaging recordings and since it is a non-invasive technique, it can be extended to different brain imaging modalities and applied at different temporo-spatial scales. In the present study, we provide a detailed description of our methodological approach, paying special attention to the theories that motivated this work. We test, for the first time, the reversibility analysis in human functional magnetic resonance imaging data in patients suffering from disorder of consciousness. We verify that the tendency of a decrease in the asymmetry of the brain signal together with the decrease in non-stationarity are key characteristics of impaired consciousness states. We expect that this work will open the way for assessing biomarkers for patients' improvement and classification, as well as motivating further research on the mechanistic understanding underlying states of impaired consciousness.

## 1. Introduction

### 1.1. Survival and metabolism from neurons to macroscopic brain dynamics

Life and survival become synonymous as soon as we delve into their very nature. Survival in life means a constant fight against a highly disordered environment. In his book *What is life? The physical aspect of the living cell*, Erwin Schrödinger famously defined survival as the circumvention of decay and of equilibrium [1]. For us, living organisms, elucidating the decay starts from our cells in the process of metabolism. This perpetual violation of thermodynamic equilibrium is scale-dependent, and different from inanimate matter, for living organisms not only arise from external forces but also from endogenous motives, driving mesoscopic mechanical forces [2–4]. This scale-dependent break of equilibrium can be addressed at the spatial and temporal levels; thus, larger biological organizations are endorsed by a departure from equilibrium processes at the molecular scale while showing apparent equilibrium in their totality [5].

There are instances of non-equilibrium in living systems. For example, some cells are able to migrate because they are propulsed by the irreversible motion of the flagella. However, this irreversible process does not mean that the cell at all scales is irreversible, the flagella works in non-equilibrium while in the intracellular medium, the cell might achieve equilibrium [3]. Measuring this intracellular equilibrium is not an easy task and great efforts have been made in detecting the breakdown of the detailed balance [2]. In the case of the brain, single neurons spiking in a network generate irreversible dynamics, given that their temporal symmetry is broken because of the process originating from action potentials. Nevertheless, this symmetry could be recovered at broader scales, where the joint activity of thousands of neurons is averaged, resulting in oscillatory local field potentials (LFPs) [6]. At the whole-brain level, healthy cognitive functions in human and non-human primates are based on a repertoire of flexible interactive neural assemblies that are spatially distributed. In this way, it seems improbable that cognition can be sustained by macroscopic brain dynamics close to equilibrium. Recent advances in this field show brain dynamics departing from thermodynamic equilibrium, brain states form a configuration space where their transition probabilities are asymmetric, there is entropy production and the produced neural activity is temporally irreversible [6–8].

### 1.2. Detailed balance and arrow of time

There is a genuine relationship between temporal reversibility and thermodynamic equilibrium. This equilibrium is built based on stochastic fluctuations that compensate over time, implying that an observer can appreciate a concrete trajectory in configuration space with the same likelihood of observing its opposite [9]. Systems at the aforementioned equilibrium are adhered to detail balance, this means that there is no net of probability fluxes in the configuration space, indicative of reversible dynamics which are associated with a null entropy production rate [6,7]. This is clearly defined in terms of thermodynamics, where a system stops to produce net entropy and becomes reversible in time [10]. Contrarily, a system presenting non-equilibrium—where the detailed balance is broken—exhibits net entropy fluxes between the underlying states and like that becomes irreversible, establishing an arrow of time [11–15]. In physics, this arrow of time is based on an asymmetry, which sets a favoured direction for the temporal evolution of a given macroscopic system (i.e. towards higher entropy states) [16]. Eddington [17] was a pioneer proposing this framework of links among non-equilibrium, irreversibility (asymmetry) and the production of entropy, leading to the arrow of time, and since then it has been extensively studied in physics and biology.

Apparently simple, the stunning concept of the arrow of time, has faced diverse approaches to study different parts of its definition and its associated subsequent terms. Clausius [18] and Carnot [19] defined the second law of thermodynamics, stating that a non-equilibrium is represented by the arrow of time which describes the irreversibility of a system. Indeed, the second law of thermodynamics can be declared by the Clausius inequality, stating that the work associated with a given process (averaged over trials) is greater than the change in its free energy. Rudolph Clausius and Sadi Carnot solved the non-trivial problem of determining the irreversibility or non-equilibrium in a system. Since its origin, the arrow of time is attached to the profound concept of causality.

Thanks to Pearl's [20] great work gathering and summarizing the information related to causality literature, we understand that in order to disentangle causal interactions in a system manipulation of the whole system under study is necessary. Any framework aiming to detect causal inferences stands on inferring causal structures that generate equivalent probability distributions, such that they are indistinguishable from observed data, requiring as a result the manipulation of the whole system in order to distinguish them. So, thermodynamics provides interesting tools for establishing the causal directionality flow of information using the reversibility and entropy concepts; however, given the concrete conditions for its analysis, causality supposes a challenge to be computed.

From the point of view of chaos theory, the arrow of time has also been investigated. Poincaré [21] made public the first characterization of chaotic motion in 1890. Long decades of work after that confirmed that a key characteristic of chaos is the extreme sensitivity to initial conditions [22]. Having been proposed that sensitivity to initial conditions, even when a classic mechanical deterministic chaotic system is *a priori* reversible, is in fact irreversible. Stated briefly, chaos hinders the establishment of computational reversibility to a large extent and so the assessment of causality.

As previously mentioned, the ideas in the present study come from physics and thermodynamics, where non-equilibrium is inherent to irreversibility [10,15] and to entropy production, originating the arrow of time [17]. We also stated that life is based on violating the detailed balance of the transitions between underlying microscopic states. In fact, the concept of the arrow of time has been largely used for non-trivial biological problems like protein folding [23]. In this way, we want to show that this key idea from physics and thermodynamics is also very useful in neuroscience.

The whole-brain scenario, where specifically brain signals are characterized by temporal asymmetries, and hence they define an arrow of time, as a result of a permanent departure from equilibrium due to internal and external stimulus. An obvious consequence of this is the increasing interest in studying entropy production among other related concepts for characterizing time reversibility in brain signals [6,7,24,25]. Research related to the cognitive effort is worth mentioning, as it identified an increase in entropy production in brain time series during demanding tasks compared to easier ones. This increase in the effort was found to be influenced by the novelty and uncertainty of the task, and the energy required to maintain or switch concentration was found to be higher in subjects with mental disorders [7,26,27]. Notwithstanding, to compute causality and hence entropy rate production is not an easy task and it is based on several assumptions that should be addressed quite carefully. In fact, we think that the arrow of time based on temporal symmetries could supply the exact right tools for capturing irreversibility and thus both external and internal drivers of a system like the brain.

### 1.3. How to detect that a system is reversible

In our daily lives, at the macroscopic level, we face many non-reversible processes. For example, when a balloon explodes because it touches something sharp. Some situations, like the one just described, represent clear illustrations of the arrow of time. If we had the chance to film the balloon exploding and we watch the reversal clip, we would be certain about what happened before and what happened after. Recalling the second law of thermodynamics, when we face an irreversible process where the system becomes disordered, it means that the total entropy production is greater than zero and, hence, the system is out of equilibrium. Contrarily, when there is no entropy production, we face a reversible system in equilibrium. We can define an irreversible process when we are able to distinguish between the time-forward and time-reversal trajectories.

The Clausius inequality states that the work associated with a process (average over repetitions) is greater than the change in its free energy. This postulate can be expressed as the average of the work associated with the forward and backward trajectories in time. Large amounts of work correspond to greater differences from the forward and backward trajectories and thus a stronger arrow of time. The systems that operate far away from thermodynamic equilibrium exchanging energy and matter with the environment are the so-called dissipative systems. Although the theories are not recent, the task of assessing reversibility is still under study. In fact, a novel measure of entropy production based on the fluctuations of dissipative systems has been developed, and besides the specific case where it was tested these ideas could be extended [28].

However, a simple, yet powerful manner of estimating the irreversibility in the brain time series is to directly assess the arrow of time encoded as asymmetries in brain signals, rather than the more intricate way of estimating entropy production. This appealing way to deal with non-equilibrium systems is creating an increasing interest in the scientific community, leading to the development of many different approaches to assessing reversibility [29]. In the present study, we will assess reversibility in whole-brain time series based on the temporal asymmetries of brain signals previously described by Deco *et al.* [8,30].

### 1.4. Measuring non-equilibrium is not trivial

The cases of a balloon exploding or a glass being shattered are clearly non-reversible processes; however, if we think of colliding billiard balls or a moving swing, the result is more ambiguous. The brain signal case is one of those processes which is not an easy task to disentangle reversible from non-reversible dynamics. For irreversible macroscopic processes like the glass or the balloon, fluctuations are trivial and the difference is obvious between the distribution of work and the arrow of time is easy to define. On the other hand, although in microscopic systems (where brain signals are included) the mean work is comparable, the fluctuations are marked and the distinction between distributions is uncertain. Given that in these situations establishing the arrow of time is harder, equally, the assessment of reversibility and equilibrium becomes arduous. Indeed, non-equilibrium states are non-stationary, meaning that reversibility fluctuates over time; this provides the second order of non-stationarity. In other words, we can assess the non-equilibrium of a system in time and how this non-equilibrium changes over time. As cited above, the level of equilibrium is linked to fluxes of transitions of different states, in our case brain states. If the transitions between brain states suppose net fluxes, our system is far from the equilibrium, and, as a consequence of broken balance, the arrow of time is defined [11,13,15].

### 1.5. Broken symmetry in conscious brain dynamics

Neural dynamics are commonly assumed as non-equilibrium processes, but macroscopic brain dynamics possess some challenges in terms of their classification [31]. Nonetheless, recent efforts have been made in order to show that the human brain does not follow detailed balance and the departures from equilibrium are task dependent. The proposed framework quantifies entropy production from functional magnetic resonance imaging (fMRI) in a reduced two-dimensional space [7]. Dissipative systems, as we mentioned earlier, evolve in a chosen temporal direction dictated by the thermodynamic arrow of time. Interestingly, our perception of time is constantly flowing from past to future, but never in the reversed manner [32]. The origin of this temporal asymmetry makes us wonder whether the conscious perception of the physical world would support that asymmetry and to what extent these asymmetries could covariate with the level of conscious awareness.

Does the asymmetry of the environment translate into temporal asymmetries of their brain dynamics representation? To what extent is the intrinsic spontaneous activity asymmetrical? More complex stimuli yield greater asymmetries or is that the case for multisensory information? Is the irreversibility of brain dynamics strictly linked to the subjective experience of the flow of time, and if so, can it be altered in different levels of consciousness? There are many and diverse questions we can ask about the characteristics of brain signal asymmetries. Indeed, recent studies have been starting to investigate some of them. Perl *et al.* [6] showed that brain states closer to equilibrium were related to a lower level of consciousness. Despite these advances, more studies need to be done to clarify the relationship between levels of consciousness and irreversible brain dynamics. As mentioned above, there are hints that make us think that at some spatial and temporal scales cognition is sustained by strong deviations from equilibrium. Having said that, it implies that unconscious brain states could obey detailed balance at large scale, although at the same time at neuronal level the homeostatic processes are irreversible. This scenario is coherent with the low entropy and complexity that the lower levels of consciousness show at large-scale brain dynamics [33–37].

### 1.6. Disorders of consciousness

It is commonly accepted that deep sleep, anaesthesia or brain damage after a wide variety of lesions producing disorder of consciousness (DOC) are characterized by a decrease or disappearance of consciousness. Both the level of wakefulness (i.e. arousal) and awareness (i.e. the content of consciousness) are assessed in clinical settings to define the state of consciousness [38]. Wakefulness is typically evaluated by eye opening, and awareness by the interaction with the environment, usually responding to elicited tasks, as a proxy for subjective experience. Investigating impairment of consciousness is essential to comprehend the neural correlates of consciousness; however, the mechanisms sustaining these levels of consciousness remain unknown. Demonstrating these underlying mechanisms is arduous since they apparently rely on a non-trivial conjunction of alternations in local dynamics and network interactions [39].

Graph theory analysis has shown that the modular and hierarchical organization of the human connectome supports robust and efficient information transmission [40,41]. Because of that, a healthy consciousness state is the result of the interplay between connectivity and dynamics favouring the coordination of brain-wide activity [42–45]. Although integration between brain areas and visiting a large repertoire of brain states are the main characteristics for a healthy brain, they are impaired in DOC patients characterized by a loss of communication at the whole-brain level [43,46–49], a loss of functional complexity [34,50] and a loss of integration [45,47,51]. A similar scenario is faced under anaesthesia; recent evidence suggests that by means of deep brain stimulation, the characteristic repertoire of brain states visited during healthy wakefulness can be restored [52]. Interestingly, functional connectivity during conscious wakefulness deviates from structural connectivity while in unconscious states the dynamics follows closer anatomical organization [43,53–55].

In the light of the recent results in characterization of non-human and human brain dynamics reversibility under different states of anaesthesia and sleep [6,8,30,56,57], we wanted to assess for the first time the level of equilibrium present in fMRI brain signals of patients suffering from DOC compared to resting state healthy controls. We expect that the level of reversibility could significatively distinguish between control group (CNT) and lower consciousness states (DOC) given that the latter group is expected to show more symmetric time series in comparison to healthy subjects. Moreover, we also expect that our method is able to differentiate between the two different groups of patients under study. The minimally conscious state (MCS) group present fluctuating but reproducible signs of consciousness [58] and the unresponsive wakefulness syndrome (UWS) group show preserved arousal but no behavioural signs of awareness [59]. The differential diagnosis of these patients is done at the bedside in clinical settings by trained physicians using Coma Recovery Scale-Revised (CRS-R). While being a useful tool to assess the state of consciousness of patients, the diagnosis can be challenging. We propose that the framework described by Deco *et al.* [30] is suitable for describing patients’ brain states in an objective and rigorous manner, paving the way towards potential biomarkers that could help with the diagnosis of those patients.

## 2. Methods

### 2.1. Participants

We included a total of 31 patients in MCS (11 females, mean age ± s.d., 47.25 ± 20.76 years), 24 in UWS (10 females, mean age ± s.d., 39.25 ± 16.30 years) and 13 healthy controls (seven females, mean age ± s.d., 42.54 ± 13.64 years) described in our previous study [60]. Trained clinicians carried out the clinical assessment and CRS-R scoring to determine their level of consciousness. Patients were diagnosed with MCS if they exhibited some behaviours that could be indicative of awareness, such as visual pursuit, orientation to pain, or reproducible command following. On the other hand, patients were diagnosed with UWS if they showed arousal (opening their eyes) without any signs of awareness (never exhibiting non-reflex voluntary movements). This research was approved by the local ethics committee Comité de Protection des Personnes Ile de France 1 (Paris, France) under the code ‘Recherche en soins courants' (NEURODOC protocol, no. 2013-A01385-40). The patients' relatives gave their informed consent for the participation of their familiar, and all investigations were performed according to the Declaration of Helsinki and the French regulations

### 2.2. MRI data acquisition

MRI images were acquired with two distinctive acquisition protocols. For the first protocol, MRI data of 21 patients and 13 healthy controls were acquired on a 3 T General Electric Signa System. T2*-weighted whole-brain resting-state images were recorded with a gradient-echo EPI sequence using axial orientation (200 volumes, 48 slices, slice thickness: 3 mm, TR/TE: 2400 ms/30 ms, voxel size: 3.4375 × 3.4375 × 3.4375 mm, flip angle: 90°, FOV: 220 mm^2^). Also, an anatomical volume was obtained using a T1-weighted MPRAGE sequence in the same acquisition session (154 slices, slice thickness: 1.2 mm, TR/TE: 7.112 ms/3.084 ms, voxel size: 1 × 1 × 1 mm, flip angle: 15°). For the second protocol, MRI data of 34 patients were acquired on a 3 T Siemens Skyra System. T2*-weighted whole-brain resting-state images were recorded with a gradient-echo EPI sequence using axial orientation (180 volumes, 62 slices, slice thickness: 2.5 mm, TR/TE: 2000 ms/30 ms, voxel size: 2 × 2 × 2 mm, flip angle: 90°, FOV: 240 mm^2^, multiband factor: 2). An anatomical volume was obtained in the same session using a T1-weighted MPRAGE sequence (208 slices, slice thickness: 1.2 mm, TR/TE: 1800 ms/2.35 ms, voxel size: 0.85 × 0.85 × 0.85 mm, flip angle: 8°).

### 2.3. Resting state pre-processing

The pre-processing of resting state data was performed using FSL (http://fsl.fmrib.ox.ac.uk/fsl) as described in our previous study [60]. Briefly, resting state fMRI was computed using MELODIC (multivariate exploratory linear optimized decomposition into independent components) [61]. Steps included discarding the first five volumes, motion correction using MCFLIRT [62], brain extraction tool (BET) [63], spatial smoothing with 5 mm FWHM Gaussian kernel, rigid-body registration, high pass filter cutoff = 100.0 s, and single-session independent component analysis (ICA) with automatic dimensionality estimation. Then, lesion-driven artefacts (for patients) and noise components were regressed out independently for each subject using FIX (FMRIB's ICA-based X-noiseifier) [64]. Finally, FSL tools were used to coregister the images and extract the time series between 100 cortical brain areas for each subject in MNI space from the Schaefer parcellation [65].

### 2.4. Assessment of reversibility in the system

**Figure 1.**
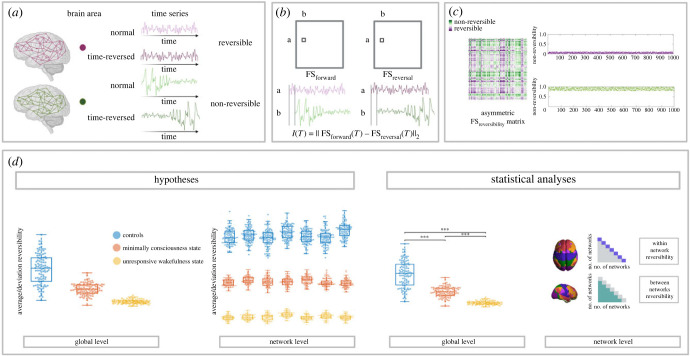
Outline of the present study, reversibility as a signature of consciousness. (*a*) Reversible and non-reversible dynamics in brain time series: we can observe an illustration of two hypothetical brain signals of fMRI from different nodes exhibiting contrary behaviours regarding their symmetry (*purple* versus *green*). In order to calculate the degree of asymmetry we must construct the time-reversed (*time-reversed*) signal from the original version (*normal*). (*b*) Computation of the functional connectivity reversibility matrix: to assess the degree of asymmetry we measure absolute quadratic difference between FS_forward_ and FS_reversal_, the pairwise comparison of the time-shifted time series. Causal interactions among regions, if they exist and have a strong time dependency (i.e. two nodes of a given network), will show a high correlation in the forward but a low correlation for the time-reversed case. The dynamical reversibility of every subject can be explained in terms of their average and deviation. (*c*) Functional connectivity reversibility matrix and subjects' levels of reversibility. The FS_reversibility_ matrix that we show in this panel is computed at every sliding window. Note that the colour purple indicates more reversible processes, as they are close to zero, and the green colour indicates more non-reversible ones. The right panels represent two contrary scenarios of dynamical reversibility of a hypothetically group of subjects. One scenario exhibits subjects in a more reversible state (*purple case*), and another where the subjects’ brain dynamics work in a more non-reversible manner (*green case*). The dots represent the absolute averaged level of reversibility in every sliding window for the whole group of subjects concatenated. (*d*) Disorder of consciousness and their level of reversibility. We hypothesize that the patients (MCS, UWS) would present lower levels of averaged and deviation reversibility compared with control group (CNT). We expect this effect to be similar at the global and network level, following the decrease of asymmetry, the decrease of consciousness (CNT > MCS > UWS). The statistical analyses at the global level will be shown as illustrated, with asterisks on top of the box plot. At the network level, for the sake of simplicity, we will show the significance between the comparison of the networks within and between conditions (CNT, MCS, UWS) in a triangle shape of *p*-value matrices; where the diagonal indicates the within-network reversibility difference (i.e. Visual versus Visual network) and the rest of the locations the between networks-reversibility difference (i.e. Visual versus Somatomotor networks).

Computing the level of non-reversibility, relies on the key idea of detecting the arrow of time through the degree of asymmetry obtained by comparing the causal relationship between pairwise time series of the forward and the artificially generated time-reversed version. More specifically, let us consider first the detection of the level of non-equilibrium (i.e. the arrow of time) between two time series *x*(*t*) and *y*(*t*) as shown in figure 1*b*, let us assume that *x*(*t*) is evolving from an initial state A1 to a final state A2, and *y*(*t*) is evolving from an initial state B1 to a final state B2, respectively. The time-reversed version of *x*^(*r*)^(*t*) (or *y*^(*r*)^(*t*)), that we call *x*^(*r*)^(*t*) (or *y*^(*r*)^(*t*)), is obtained by flipping the time ordering, (i.e. by ordering the time evolution of *x*^(*r*)^(*t*) (or *y*^(r)^(*t*)) as the inverted sequence determined by initial state A2 to a final state A1 (or initial state B2 to a final state B1). The causal dependency between the original time series *x*(*t*) and *y*(*t*) are measured through the time-shifted Pearson correlation. For the forward evolution the time-shifted correlation is given by

$$c_{\mathrm{forward}}(\Delta t)=\left\langle x(t),y(t+\Delta t)\right\rangle ,$$

and for the reversed backward evolution the time-shifted correlation is given by the pairwise level of non-reversibility, i.e. the degree of temporal asymmetry

$$c_{\mathrm{reversed}}(\Delta t)=\left\langle x^{\left(r\right)}(t),y^{\left(r\right)}(t+\Delta t)\right\rangle ,$$

capturing the arrow of time, is given consequently by the absolute difference between the causal relationship between these two time series in the forward and reversed backward evolution, at a given shift Δ*t* = *T*, i.e.

$$I_{x,y}(T)=|c_{\mathrm{forward}}(T)-c_{\mathrm{reversed}}(T)|.$$

We keep the correlations with their signs and compute the absolute difference as we are interested in the magnitude of the change in the asymmetry. After computing the autocorrelation for all regions, subjects and conditions, we observed that a sufficient decay occurs when *T* = 1. This value *T*, reflects the temporal domain where we assess our results, adjusting this parameter will allow us to find different results at different timescales. Of note, this *T* represents the amount of timepoints shifted of the discretized signal, meaning that the real-time association will depend on the time resolution of the acquired data. This exploration might seem incomplete without referencing the INSIDEOUT method. Therefore, it is important to mention that in previous work using INSIDEOUT, a fine-tuning of *T* was performed following the analysis of the autocorrelation [30]. However, it should be noted that the sample rate of the signals in that case was relatively short (less than 1 s), whereas in our study using fMRI data, the sample rate is approximately 2 s, which might be considered too high for such fine-tuning.

The level of non-reversibility/non-equilibrium for the multidimensional case of whole-brain analysis can be easily generalized by defining the forward and reversal matrices of time-shifted correlations. Let us denote with *x_i_*(*t*) the forward version of a multidimensional time series reflecting the dynamical evolution of the variable describing the system. The sub-index *i*, in this case, indicates the distinct dimensions of the dynamical system. Let us denote with $x_{i}^{\left(r\right)}(t)$ the corresponding reversed backward version. The forward and reversal matrices expressing the functional causal dependencies between the different variables for the forward and artificially generated reversed backward version of a multidimensional system are given by

$${\mathrm{FS}}_{\mathrm{forward},ij}(\Delta t)=-\frac{1}{2}\log\left(1-{\left\langle x_{i}(t),x_{j}(t+\Delta t)\right\rangle }^{2}\right),$$

$${\mathrm{FS}}_{\mathrm{reversal},ij}(\Delta t)=-\frac{1}{2}\log\left(1-{\left\langle x_{i}^{\left(r\right)},(t),x_{j}^{\left(r\right)}(t+\Delta t)\right\rangle }^{2}\right),$$

respectively. The FS functional causal dependencies matrices are expressed as the mutual information based on the respective time-shifted correlations. The level of non-reversibility is given by the quadratic distance between the forward and reversal time-shifted matrices, at a given shift Δ*t = T*. In other words, the level of non-reversibility/non-equilibrium in the multidimensional case is given by

$$I=\;\|\;FS_{\mathrm{forward}}(T)-{\mathrm{FS}}_{\mathrm{reversal}}(T){\|}_{2},$$

where the notation *||Q||*_2_ is defined as the mean value of the absolute squares of the elements of the matrix *Q*. In other words, if we define a difference matrix FS_reversibility_ in the following way

$${\mathrm{FS}}_{\mathrm{reversibility},ij}={\left({\mathrm{FS}}_{\mathrm{forward},ij}(T)-{\mathrm{FS}}_{\mathrm{reversal},ij}(T)\right)}^{2}.$$

The matrix FS_reversibility_ is thus a matrix whose elements are the square of the elements of the matrix (FS_forward_ (*T*) − FS_reversal_ (*T*)) where for each pair, the level of non-reversibility as measured by the squared difference. Thus, *I* is simply the mean value of the elements of FS_reversibility_. In summary, we are able to reduce the mutual information (FS), forward and reverse cases, based on the respective time-shifted (*T*) correlations into a single value corresponding to the mean of the FS_reversibility_ matrix that defines the degree of asymmetry of the time series. However, this process could be done iteratively by dividing the time series into a subset of time points, i.e. a sliding window of length *W*, resulting in a dynamic characterization of the asymmetry within a subject. Every sliding window (*W*) is described by the mean of its corresponding FS_reversibility_ matrix. For instance, every subject is described by a set of *N* reversibility values (mean of FS_reversibility_ matrix) being *N* the number of total sliding windows, depending on the length of the time series and the window size (*W*). We computed the FS_reversibility_ over all participants and all sliding windows for each condition. For the global level of asymmetry, we computed for each sliding window the degree of asymmetry as the mean value of the elements of the FS_reversibility_ matrix. Each subject is characterized as the average reversibility values and their standard deviation, later on called deviation reversibility. For the network level first, we identified the nodes that belong to the same network, being the seven resting state networks based on the 100 nodes of the Schaefer parcellation [65]. We followed the same steps for each network, starting with defining it as a subset of nodes. Firstly, we applied time-shifted (*T*) Pearson's correlation to both forward and reverse cases of the original signals on the corresponding nodes, then calculated mutual information (FS) among them, and finally took the mean of the FS_reversibility_ matrix. In the pairwise analysis, the size of the FS_reversibility_ matrix is *N × N*, where *N* is the total number of areas. However, in the network analysis case, the size is variable depending on the number of nodes from the networks under comparison. Like that, we are able to summarize the asymmetry within or between networks with a single value of reversibility. As stated before, we can iteratively perform these computations over sliding windows in the same fashion resulting in a dynamical network analysis of reversibility within a subject. Briefly, we computed the pairwise comparison of the nodes corresponding to a given network with the rest of the networks and itself and then averaged their causal interaction in a 7 by 7 diagonal and subdiagonal matrix of differences, at this level we also assessed the deviation reversibility.

### 2.5. Statistical analysis of the results

All statistical analyses of the data conducted here used the standard statistical Mann–Whitney *U* test, also known as the Wilcoxon rank sum method [66], and false discovery rate (FDR) Benjamini–Hochberg [67] correction for multiple comparisons (as specified in the text). The data are available upon request.

## 3. Results

### 3.1. How we measure symmetry in brain signals

In general, there are two main approaches being developed in the assessment of reversibility. Reversibility or in general time symmetry can be approached as a geometric characteristic of a stochastic process or assuming that there are some physical source sustaining that process [29]. The present analysis belongs to the latter assumption, where its ideal last step would be the computation of entropy production. Zanin *et al.* state that no method is ideal and *one-size-fits-all* is not the case for reversibility studies; moreover, the conclusion inferred from the results of this kind of analysis should be taken cautiously.

Figure 1 shows a schematic of the outline executed in the present reversibility analysis. In figure 1*a*, brain dynamics captured by fMRI reveal a macroscopic system governed by the asymmetry of the arrow of time. Yet, these signals display a variety of behaviours regarding the mentioned asymmetry. As an example of this, we can observe an illustration of two hypothetical brain signals from different nodes exhibiting contrary behaviours regarding their symmetry. The time-reversed signal (*green*) is constructed as the specular image of the original signal (*purple*) read from time 0 to time T, as indicated with the arrow. On the other hand, the green signal depicts a clearly non-reversible, hence asymmetric time series. In this case, it is quite easy to differentiate between the normal and time-reversal signals.

Normal and time-reversed whole-brain fMRI signals are used to assess the reversibility of the brain both at global and network level, as is displayed in figure 1*b*. In order to create a direct link between our assessment of non-equilibrium/non-reversibility and broken detailed balance, we measured the asymmetry of the time-shifted functional connectivity. We perform the pairwise correlation of the time series shifted in time (τ=1) for both the normal and time-reversed signals. Like that we construct the functional shifted in time correlation matrices FS_forward_ and FS_reversal_. Then, we compute the difference between matrices and obtain the absolute value as the reversibility level. If two brain nodes present a high temporal dependency, the subtraction of the forward and reverse correlation will be large. Instead, two brain regions with poor temporal dependency will show zero or close to zero reversibility. We calculate the level of reversibility as the absolute quadratic difference between the time-shifted matrix FS_forward_ and FS_reversal_, averaged over all pairs of nodes. Shorter distances suppose that the whole-brain is working near reversibility, such that larger distances depict the brain working away from equilibrium. We selected a size of 90 time points for creating sliding windows. We divided the whole time series into chunks of 90 timepoints that overlapped 85 points. We computed the FS_reversibility_ matrix for every sliding window in order to get a dynamical information of reversibility and hence, a second order non-stationary value. The dynamical reversibility of every subject can be explained in terms of their average and deviation, giving us a more comprehensive and deep understanding of their brain dynamics.

Irreversibility is associated with breaking the detailed balance, as shown in figure 1*c*. Specifically, the level of asymmetry of the computed FS_reversibility_ matrix is a proxy for the non-equilibrium of the system. More asymmetry corresponds to more irreversibility. We represented in purple the values closer to 0 and in green the values that fall far away from zero. The FS_reversibility_ matrix is asymmetric itself because the correlation difference varies depending on the inherent temporal causal structure of the data. In addition, the right value represents an illustration of two different theoretical groups of subjects. The dots represent the average value of reversibility obtained per subject at every sliding window, the subjects are concatenated in time. The upper plot (*purple dots*) represents a group of subjects with low average reversibility and the lower plot (*green dots*) represents a group with higher average reversibility closer to one. The purple group presents less variability, while the green group has a higher deviation. The reversibility dynamics of the purple group are closer to the equilibrium and hence, more symmetrical than the green group which represents a greater departure from the equilibrium sustained by asymmetrical brain signals.

The hypothesis of this work is based on the proposed framework and with it we want to demonstrate that the DOC groups (*red and yellow*) present less asymmetrical dynamics compared to the CNT group (*blue*) and hence, they work closer to the equilibrium, as illustrated in figure 1*d*. We expect that equally average and deviation values of reversibility show a decrease from CNT to UWS group, representing the MCS an intermediate state of consciousness compared to the other two groups. This result is graphically presented in the *hypothesis section* by means of *global* and *network level* results. We hypothesize that both at the global and network level the differences will be present in a general way, showing a coherent descent between the two levels under study.

The results obtained from the reversibility analysis based on brain signal symmetry of DOC patients and CNT subjects are split into global and network levels for their study.

### 3.2. Global level results

**Figure 2.**
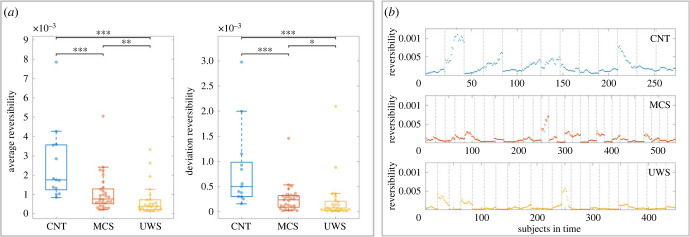
Global level results. (*a*) At the left panel, we can observe the distribution of the average reversibility in every group, composed of the averaged reversibility of every subject defined by a set of 90 time points sliding windows, a total 21 or 17 sliding windows depending on the length of the time series. At the right, we find three distributions of the standard deviation of the reversibility of every subject at each condition, expressing the dynamics of the reversibility over time. As can be seen the average reversibility of the CNT group is significantly greater than the other two DOC groups, both MCS and UWS. Also we can see that among these two latter groups there exists a significant difference. The standard deviation of the reversibility shows in agreement with the previous finding, being greater for the CNT group and decreasing as the level of consciousness, followed by MCS and UWS, respectively. Suggesting that the CNT group is not only, on average, further from the equilibrium, but also that the variability between reversible and non-reversible processes is increased compared to the DOC groups. The level of significance after FDR correction between conditions is indicated schematically (*p < 0.005, **p < 0.01, ***p < 0.001). (*b*) In this section, we graphically show the absolute averaged values of reversibility at every window for every subject concatenated (delimited with vertical lines) and divided per conditions. The upper panel represents the CNT group which exhibits a greater variability and values further from zero than other groups. The middle panel describes the MCS group, characterized by lower absolute values, which are closer to zero compared to the CNT group and dressed variability as well. Last, the inferior panel corresponds to the UWS group, this group possesses the least variability and their reversibility values are the closest to zero, representing the most extreme case of reversible state within the three conditions.

In figure 2, we summarized the information at the subject level divided by the three conditions under study. Specifically, in figure 2*a*, we can appreciate the distribution of reversibility per group in terms of their average and stationarity (deviation). Note that the axes from the average reversibility plot and the deviation reversibility plot differ as the average reversibility values are greater than the latter ones.

#### 3.2.1. Average reversibility

As we can observe, in blue the CNT group (reversibility = 0.0018 ± 0.0019, *n* = 13), in red the MCS (reversibility = 7.64 × 10^−4^ ± 9.92 × 10^−4^, *n* = 31) group and in yellow the UWS group (reversibility = 3.89 × 10^−4^ ± 8.21 × 10^−4^, *n* = 24). The CNT group presents a significantly greater distribution of reversibility compared to both MCS (***p < 0.001) and UWS (***p < 0.001) groups. The dots in the box plot represent the subject-level average value of reversibility for their sliding windows time-series analysis. In the same way, the MCS group in red presents a significantly greater distribution compared to the yellow UWS group (**p = 0.0098). As we expected the CNT group, which in this comparison represents the greatest level of consciousness, is associated with higher asymmetry as their level of reversibility is further from zero than the DOC groups. The same can be said about the DOC groups, a clear difference in terms of reversibility exists between the MCS group and the UWS group, explained by the fact that the UWS group is being sustained by more symmetrical brain signals compared to the more conscious MCS group. The statistical comparisons are computed by means of the Wilcoxon rank sum test and after corrected for multiple comparisons by the FDR Benjamini–Hochberg.

#### 3.2.2. Deviation reversibility

The stationarity of the reversibility is computed as the standard deviation of the reversibility values given at every sliding window per subject. As we can appreciate, the CNT in blue (deviation reversibility = 5.0279 × 10^−4^ ± 8.1586 × 10^−4^, *n* = 13), the MCS group in red (deviation reversibility = 2.37 × 10^−4^ ± 2.75 × 10^−4^, *n* = 31) and the UWS group in yellow (deviation reversibility = 7.45 × 10^−5^ ± 4.41 × 10^−4^, *n* = 24). The CNT group is the most non-stationary group compared with the MCS group (***p < 0.001) and UWS group (***p < 0.001). However, in this situation, although significant, the distance between the MCS and UWS groups is smaller (*p* = 0.0408), indicating that the DOC groups are characterized by a more stationary reversibility regime compared to the healthy resting state. The statistical comparisons are computed by means of the Wilcoxon rank sum test and after corrected for multiple comparisons by the FDR Benjamini–Hochberg.

#### 3.2.3. Sliding windows at subject level

In figure 2*b*, we can observe three different plots associated with the three different levels of consciousness. Ranked from top to bottom, the higher level of consciousness is the CNT group followed by MCS and the lower state of consciousness UWS group. In each of these plots, we can appreciate the reversibility (*y*-axis) in time per subject (*x*-axis), as a concatenation of all of them in each condition, to create a visual illustration of the previous distributions described. The dots represent the averaged reversibility obtained from the FS_reversibility_ matrix at every sliding window, a total of 21 or 17 (depending on the time-series length) per subject. At a glance, we can note that the CNT group is the one showing higher and more diverse reversibility values. On the other hand, the MCS and UWS are characterized by lower and more stable levels of reversibility compared to the CNT. Indeed, the MCS presents more variability within and between subjects compared to the UWS, where most of them show almost the same value of reversibility within and between subjects, with the exception of three subjects that represent the three outliers of the previous box plots.

### 3.3. Network level results

**Figure 3.**
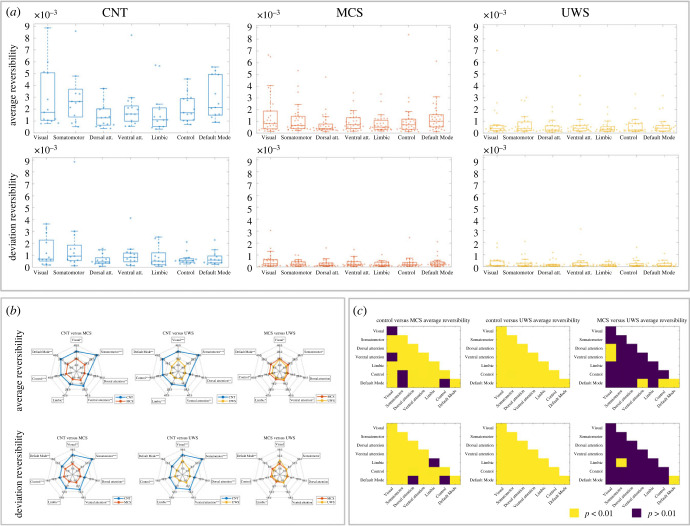
Network level results. (*a*) This section shows the average and deviation reversibility of the seven resting state networks of the parcellation used (see Methods). *The upper line of plots* summarizes the results for the average reversibility at the network level per condition. The CNT group shows in general a higher level of non-reversibility compared to the other DOC groups. The MCS and the UWS possess mean values of reversibility closer to zero. However, the UWS falls behind the MCS as expected. The Somatomotor network followed by the Visual network represents the highest reversibility on average among the networks for the CNT. However, this trend is inverted for the DOC where the Visual network is on average the furthest to zero followed by the DMN in the case of the MCS and by the Ventral attention for the UWS condition. *The inferior line of plots* shows the standard deviation of the brain regions corresponding to every network at each condition. The CNT group shows the highest variability of reversibility compared to the other two groups. The DOC groups show a marked decrease in deviation reversibility; in this case, the MCS group and the UWS group are similar. Although difficult to see at a glance, the MCS group is still the middle point between the UWS condition and the CNT group. In the CNT group, on average, the Somatomotor network is the most variable one followed by the Visual network in agreement with the average reversibility. In the DOC groups the Visual network is the most variable followed by the DMN in the MCS case and followed by the Ventral attention in the UWS condition. (*b*) This section shows a graphical representation of one to one comparison between groups of the distributions previously shown in (*a*). The upper line of plots corresponds to the average reversibility, the inferior line of plots represents the deviation reversibility. Note that the values are multiplied by 10^4^ for the sake of clear visualization. The level of significance after FDR correction between conditions is indicated schematically with the name of the network in bold and asterisks for the level of significance (*p < 0.005, **p < 0.01, ***p < 0.001). (*c*) The level of significance after FDR correction between conditions binarized at *p* < 0.001.

In figure 3, we summarize the average and deviation reversibility results at the network level divided into the three groups under study (figure 3*a*) and the statistical comparisons between them (figure 3*b*,*c*).

Figure 3*a* shows similar box plots to the second figure. Now the difference is that in each condition plot, in the *x*-axis there are seven resting state networks from the Schaefer 100 nodes parcellation [65]. The results are equally distributed in average (upper line of plots) and deviation reversibility (lower line of plots).

#### 3.3.1. Average reversibility

The three first plots from left to right represent within network average reversibility for the CNT, MCS and UWS groups. As we can observe, the CNT group, replicating the global level results, shows the greatest values of reversibility in a general manner compared to the DOC groups. In addition, the DOC groups are equally divided in the more asymmetrical MCS group (higher level of consciousness) and UWS (lower level of consciousness) group as the least asymmetrical, also in a general manner. Of note, among all the RSN, the CNT group shows that the Somatomotor is the network that possesses, on average, a greater value compared to the DOC groups where instead the Visual network possesses greater value. Interestingly, the Visual network follows the Somatomotor in the CNT group. As there is a great difference between CNT and the DOC groups in order to maintain the same axis for a clear visualization of the distribution in each group and avoid the collapse of box plots we discarded an outlier from the CNT group (*subject n = 2*) in the Somatomotor network (*reversibility value = 0.0186*). We did the same with another outlier from the UWS group pertaining to the Visual network (*subject n = 13 reversibility value = 0.0114*). The statistical analysis without the outliers mentioned did not produce any difference from the conjunct result present here.

#### 3.3.2. Deviation reversibility

In the lower line of plots with the same disposition as that previously described, we find the distribution of within networks stationarity in terms of their reversibility for each condition. In line with the results of the global level, the CNT group shows the highest variability in their reversibility level compared with the DOC groups. In this case, we can clearly see how both DOC groups show conjunctively a decreased non-stationary behaviour, apparently a shared characteristic of lower states of consciousness. However, there is still a tendency to conserve some non-stationarity in the MCS compared to the UWS group, specifically there are significant differences between the two networks. The network with the highest deviation on average for the CNT group is Somatomotor and for MCS and UWS groups, the Visual network, suggesting a coherent behaviour between average and deviation reversibility.

For the sake of simplicity, in figure 3*b* we aim to show a visual representation of the three different comparisons one at a time.

#### 3.3.3. Average reversibility

The upper line of plots represents the average reversibility within networks. The first spider plot represents the average reversibility per network for the comparison of CNT and MCS groups. The numbers in the plot represent the value of the reversibility multiplied by 10^4^ because of the legibility of the plot. In bold, we represent the significant differences with asterisks indicating their level of significance after the Wilcoxon rank sum test and posterior FDR correction. All the networks present a significant difference compared to the MCS group, the same and with a larger distance occurs with the second plot CNT versus UWS. The last comparison between the MCS and UWS groups is significant for all the networks except for the dorsal attention. Thanks to this plot we can observe easily the most irreversible networks in each case previously mentioned. The distance from the most irreversible network (Somatomotor) in the CNT group is greater than the distances found between the Visual network and the rest for the DOC groups. The statistical comparisons are computed by means of the Wilcoxon rank sum test and after corrected for multiple comparisons by the FDR Benjamini–Hochberg. (CNT versus MCS: *FDR corrected p-values* (*Vis. p = 0.0156, Som. p < 0.001, Dors. att p = 0.0026, Vent. att p = 0.0031, Lim. p = 0.0167, Con. p < 0.001, DMN p = 0.0019*). CNT versus UWS: *FDR corrected p-values* (*Vis. p < 0.001, Som. p < 0.001, Dors. att p < 0.001, Vent. att p < 0.001, Lim. p < 0.001, Con. p < 0.001, DMN p < 0.001*). MCS versus UWS: *FDR corrected p-values* (*Vis. p = 0.0346, Som. p = 0.0225, Dors. att p = 0.0631, Vent. att p = 0.0235, Lim. p = 0.0171, Con. p = 0.0346, DMN p = 0.0032*).)

#### 3.3.4. Deviation reversibility

The lower line of plots represents the deviation reversibility and the individual comparisons following the previous scheme. With this representation, we can easily see the differences already mentioned in the box plot. Specifically, we can observe that the only two networks that are significantly different in terms of their stationarity are the Default Mode network (DMN) and the Limbic network. The statistical comparisons are computed by means of the Wilcoxon rank sum test and after corrected for multiple comparisons by the FDR Benjamini–Hochberg. (CNT versus MCS: *FDR corrected p-values* (*Vis. p = 0.0040, Som. p < 0.001, Dors. att p = 0.0014, Vent. att p = 0.0010, Lim. p = 0.0020, Con. p < 0.001, DMN p = 0.0022*). CNT versus UWS: *FDR corrected p-values* (*Vis. p < 0.001, Som. p < 0.001, Dors. att p < 0.001, Vent. att p < 0.001, Lim. p < 0.001, Con. p < 0.001, DMN p < 0.001*). MCS versus UWS: *FDR corrected p-values* (*Vis. p = 0.0681, Som. p = 0.1165, Dors. att p = 0.1088, Vent. att p = 0.0946, Lim. p = 0.0196, Con. p = 0.1126, DMN p = 0.0083*).)

Apart from the within-network analysis that we already described, we also perform between-networks analysis and compared among conditions. In figure 3*c*, we can appreciate a binarized (*p* = 0.01) summary of the significant comparisons among conditions. If we recall figure 1*c*, the diagonal of the triangle shape plot corresponds to the within-network comparison already shown in the previous spider plots. The rest of the figure corresponds to the combinatorial comparisons of the different networks as indicated with their names on the axes. Following the scheme of average/deviation reversibility analyses, we distributed the comparisons in two lines, being the upper line of plots dedicated to average reversibility and the lower part to deviation reversibility plots. The behaviour is similar for both average/deviation reversibility situations, in general, CNT versus UWS is significant in any network comparison due to the most extreme states of consciousness analysed here. This is followed by the CNT versus MCS which show weaker network comparison, such as within the Visual, Ventral attention–Visual, Control–Somatomotor and DMN–Somatomotor (average reversibility) and within Limbic, DMN–Dorsal attention and DMN–Control (deviation reversibility). This is expected as MCS is closer than UWS in terms of the level of consciousness. Also, the MCS versus UWS comparison is expected to present the least differences because they are both groups of patients with DOC. Interestingly, some network comparisons that possess a *p* < 0.001 between MCS and UWS, but they do not for CNT versus MCS, at the average reversibility. For instance, Ventral attention–Visual and DMN–Control. Finally, it can be said that there are less comparisons that survive after the binarization in the MCS versus UWS deviation reversibility case, illustrating the common DOC characteristic of gaining stationarity.

## 4. Discussion

Computational neuroscience has recently faced the emergence of promising measurements based on reversibility and entropy rate quantification, motivating us to pursue the present work. Specifically, we wanted to characterize, from a temporal asymmetries perspective, patients suffering from DOC to establish a relationship between reversibility and level of consciousness. Thanks to the INSIDEOUT framework's ideas, we successfully demonstrate that the level of reversibility could represent a signature of consciousness, as temporal asymmetries significantly differ between the healthy resting state, MCSs and unresponsive wakefulness states. Indeed, patients showed a scale-dependent decrease of average reversibility based on their level of consciousness compared to controls and a joint decrease of non-stationarity for both groups of patients compared to the healthy resting state.

The INSIDEOUT framework, originally proposed by Deco *et al.*, is inspired by the ideas of Prof. Buzsaki, who defined the brain as a complex system that self-organizes and constrains its own activity, rather than being highly dependent on stimuli or sensations. However, it should not be confused with the use of the term ‘inside out’ by Prof. Buzsaki in his book *The brain from inside out* [68]. The INSIDEOUT framework aims to measure the alteration in the hierarchy of causal interactions of brain activity in different brain states. This methodology is based on the concept of the arrow of time and its link with non-equilibrium and time asymmetry [30]. These ideas from statistical physics are applied to brain signals to characterize the level of reversibility/non-equilibrium [17]. Specifically, the measurements are performed directly from the empirical data without any underlying model assumptions, using time-shifted correlations. INSIDEOUT framework is flexible and versatile because it can be applied at different temporo-spatial scales and it is not restricted to the fMRI dataset as presented here, being easily extended to other neuroimaging modalities such as magnetoencephalography (MEG), electroencephalography (EEG), LFP or, as already proved, electrocorticography (ECoG) [6,8,30,57].

Recently, there have been many attempts to quantify the non-equilibrium in brain time series using methods like complexity metrics or by means of entropy production rate, all of which come with drawbacks [6,7,15,69–71]. For example, entropy measurements require an estimation of transition probabilities in the state space of the system, certain assumptions (i.e. Markovian chains) and sufficiently large time series to be studied, moreover, entropy estimates might be inaccurate if our analysis is based on partial information [16,29,57,72]. Bearing in mind the challenge that entropy production analysis entails, we decide to perform a simpler, yet, quantitative, versatile and flexible measurement of reversibility to our data, based on the INSIDEOUT framework. Nonetheless, the present study represents just a vertex in the complex net of reversibility analysis and we are highly encouraged to learn from different perspectives about irreversibility in brain dynamics. Only by means of such an integrative approach will we be able to get a coherent view of reversibility, helping us to disentangle the origin, causes and consequences of this phenomena.

Our results indicate a clear distinction between CNT and DOC groups, where the latter are described by more symmetrical brain signals. At the *global level*, the average reversibility distribution showed a decreased non-equilibrium from CNT to UWS, with the MCS being the intermediate non-equilibrium stage corresponding to the intermediate consciousness state as shown in the three studied groups. This result is consistent with previous analyses of entropy production and reversibility in ECoG non-human primate and human fMRI data that showed proximity to equilibrium for states of reduced consciousness (sleep and anaesthesia) compared to conscious wakefulness [6,30]. The non-stationarity, or standard deviation, of the reversibility showed for the patients the same decrease compared to healthy subjects, suggesting a common signature for both impaired consciousness states (MCS and UWS). Interestingly, De La Fuente *et al.* built a machine-learning classifier that discriminated reversibility in brain signals finding the most important features of healthy wakefulness transitions between slow (≈20 Hz) and fast frequencies (greater than 40 Hz), considered as the main contributors to the temporal asymmetry. These transitions can also be seen as a non-stationary process of conscious wakefulness, supporting our findings [57]. The dynamic regime of the brain is key to understanding brain states in health and disease [73]. In fact, it has been shown that unconscious brain states are dominated by synchronous and less asymmetrical activity, in both macroscopic and microscopic scales [74–80], whereas conscious states are characterized by asynchronous dynamics [76]. Our global level results, according to the revised literature, suggest that impaired consciousness states are characterized by more symmetrical and stationary regimes compared to healthy resting state controls which showed more asymmetric non-stationary time series.

At the network level, we further confirm this general perspective. Within condition analysis of network reversibility, both average and standard deviation values did not reflect any change, suggesting that the whole-brain is working under a different regime and this trend is not originated by a single network but it is rather a general behaviour of brain signals . However, within condition, network reversibility showed differences when the subjects were facing distinct tasks (i.e. memory, motor, emotional tasks) [8] or situations (i.e. eyes closed versus eyes opened) [25]. In fact, higher levels of non-equilibrium were found while performing tasks compared to resting state both in sensory and higher cognitive associative areas [8]. Our results at the network level reveal a similar tendency, being more irreversible in the CNT subjects at both global and network levels, as well as, both average and deviation analysis compared to the MCS and UWS subjects. Also, we face the same escalation from higher (CNT) to lower level of consciousness (MCS > UWS) for the average and a less clear distinction for the deviation reversibility among the two DOC groups. In light of recent discoveries, the impaired consciousness states follow the lack of asymmetrical content in brain signals that characterizes neurological diseases [8,25]. Although a general lower level of non-equilibrium has been found, specific brain areas can act differently according to the disease or the symptomatology under study. For example, attention deficit hyperactivity disorder and bipolar patients presented decreased average non-equilibrium in some brain areas and increased in the areas concerning symptomatology-related networks; moreover, patients were characterized by an increase in non-stationarity, exemplifying the diversity in reversibility for a given disease [8]. In regard to concrete networks that appear as the most irreversible per condition, the CNT group shows the Somatomotor followed by the Visual network as the top irreversible networks both by average and deviation values. The DOC groups, on the other hand, present the Visual network as the top average and deviation irreversible network. The decrease in the irreversibility of the Somatomotor network in DOC groups can be related to the lack of movement and response to stimuli that often characterize impaired consciousness states [81]. In fact, in animal experiments that have suffered a removal of thalamocortical inputs, brain dynamics are regular in the absence of extrinsic stimulation and they unfold near the equilibrium [82,83]. However, our data are only representative of cortical regions and further work should be done including subcortical areas so they can be contrasted with the results shown here. Also, the Visual network is the most irreversible for the DOC groups. Although unexpected, recent studies have demonstrated that the Visual network can be partitioned into multiple areas due to diverse genetic programmes, with some of these areas being implicated in higher associative cognitive function [84,85]. Another interpretation could be that certain connections with the subcortical areas remain and the activation of the Visual network was related to some unconscious emotional processing [86–88]. The joint differential activation of the Visual, Somatomotor and the DMN, following the decrease in the level of consciousness, is certainly a key characteristic that has been discovered through different methodologies in previous experiments [60,89–92].

The origin of reversibility in neural time series remains to be uncovered. Is the irreversible nature of the stimulus the main creator of this signal asymmetry? There are different non-exclusive theories that propose explanations for this fact. Regarding macroscale brain time series, brain activity is inherently asymmetric, independently of how sensory stimuli are ordered in time. In fact, Lynn *et al*. [93,94] demonstrated, using tools of statistical physics, that the interplay between retinal neurons is the main contributor to non-equilibrium, even when a reversible stimulus is shown. A counterintuitive finding is that subjects watching a movie presented more symmetrical brain dynamics than in resting state [95]. An open question remains: how does the complexity of the stimulus reflect on brain dynamics? A recent inspiring study in music complexity could convey a possible answer by applying the same reversibility measures to musical compositions as well as to the brain signals of subjects hearing that music [96]. On the other hand, the asymmetry in brain signals could arise in neuroimaging techniques such as fMRI, given that non-reversible physiological processes (i.e. the haemodynamic response) intervene between neural responses and recorded signals [97]. From an ontogenetic perspective, the brain is developed within a temporally asymmetric environment, therefore, even in unconscious states, the emergence of intrinsic activity patterns could present this asymmetry, suggesting a baseline property of constant firing near functional patterns for brain signals [98–100]. Other interpretations of brain temporal asymmetry advocate for tasks related to facts such as memory consolidation and learning activity [101] or constant prediction of consequences and modelling of the environment [102–104].

The accepted theories of consciousness, including global neuronal workspace theory (GNWT) and integrated information theory (IIT), agree that dispersed brain activity and coordinated interplay are necessary for healthy cognition and consciousness [35,105,106]. GNWT views consciousness as a virtual place accessible to all brain mechanisms, while IIT focuses on the intrinsic properties of consciousness and the physical substrate that explains it. Other theories advocate for a shared dynamical view of consciousness, where the body and interoception mechanisms play a crucial role [107,108]. Recent attempts to combine GNWT and IIT through the free energy principle and active inference framework define consciousness as a dynamic core of integrated information occurring thanks to highly connected networks or hubs, allowing body-centric experience through phenomenal binding and executive control [109,110]. But, how does the brain self-organize to generate the dynamic requirements proposed in these theories? Our results suggest that asymmetric non-stationary brain signals may be relevant to conscious states, while impaired consciousness states may result in more symmetrical dynamics closer to equilibrium.

The inference from the reversibility analyses can be tempting, leading some authors to state that the reversibility in brain time series can: translate the arrow of time of the driving environment into brain dynamics [30], be the tangible representation of the subjective experience of time [57], or be the reflex of the inner time of a given subject [111]. Here, we simply state that reversibility analyses can be used as a good proxy to quantify the difference between levels of consciousness. Notwithstanding, any of the proposed theories can be right or even an ensemble of them distributed in different time and spatial scales. In any way, we are already convinced that the proposed reversibility framework as well as entropy production analyses are motivating the neuroscience field and we augur new discoveries under this branch of statistical physics.

## 5. Future directions

A great advantage of this analysis is that we managed to find differences among groups unveiling the differences between levels of consciousness despite attending to the origin of the disorder or concrete lesions. Although encouraging, this study has some limitations that need to be acknowledged. The reversibility results obtained in this study exhibit heterogeneity within patients of the same level of consciousness category, making a direct interpretation of them intricate. The reversibility values at the network level are influenced by the location of brain damage, with many lesions involving extensive white or grey matter areas. These lesions can be correlated with the associated DOC category, and thus, a more neurologically informed analysis could provide valuable insights at the individual level explaining differences among chronic and acute patients. Additionally, blood samples and psychological tests could be used as regressors to assess the extent to which they account for variability in our reversibility results. The relationship among measures could also be used to unveil a mechanistic understanding of the heterogeneity observed. Nevertheless, significant challenges still remain, as the number of subjects per condition is not high, and further analysis warrants the inclusion of the subcortical and cerebellar areas.

Future work should include simultaneous analysis of temporal symmetry of endogenous and stimuli-evoked brain activity to characterize the propagation of the stimulus and unveil the mechanistic underlying DOC. A good attempt, for example, could integrate the present framework with a whole-brain model in a turbulent regime that helps us understand how brain dynamics organize in light of the second law of thermodynamics. For example, a good direction could integrate the present framework with a whole-brain model in a turbulent regime to understand how brain dynamics organize in light of the second law of thermodynamics. Furthermore, as shown in a recent publication, DOC presents a diminished diversity of spatial harmonic patterns compared to healthy controls, thus another approach of studying levels of consciousness could be to understand whether this decrease in spatial diversity is reflected in temporal terms as well and in agreement with our current work [112]. Ultimately, if we were able to detect common features, based on the proposed method, in patients from DOC, we could build a support vector machine classifier and further improve it including metainformation of neuroimaging from patients that progress to a favourable state. As a result, we could use all this information about reversibility and combine it with other physiological measurements to generate a battery of biomarkers as a diagnostic and prognostic tool, helping doctors in clinical practice and improving the life of the patients suffering from DOC.

## Data Availability

This article has no additional data.
